# Anthocyanins improve liver fibrosis in mice by regulating the autophagic flux level of hepatic stellate cells by mmu_circ_0000623

**DOI:** 10.1002/fsn3.3281

**Published:** 2023-05-11

**Authors:** Jinhui Du, Likun Liu, Haiqing Fan, Yue Yu, Yilin Luo, Fang Gu, Hui Yu, Xin Liao

**Affiliations:** ^1^ Department of Medical Imaging The Affiliated Hospital of Guizhou Medical University Guiyang City China

**Keywords:** anthocyanins, autophagy, circRNA, hepatic stellate cells, liver fibrosis

## Abstract

Liver fibrosis is a key step in the progression of various chronic liver diseases to liver cirrhosis and even liver cancer, it is also an important link affecting prognosis. Therefore, this study aimed to investigate the therapeutic effect of anthocyanins on liver fibrosis and the molecular mechanism of mmu_circ_0000623 in anthocyanin therapy. In this study, CCL_4_ was used to build a mouse liver fibrosis model, and the treatment groups were treated with 100 and 200 mg/kg of anthocyanins daily by gavage. Liver fibrosis indicators, macrophage polarization markers, and liver injury markers were further detected by real‐time quantitative PCR (qRT‐PCR), western blotting (WB), and enzyme‐linked immunosorbent assay. Morphological verification of liver injury in different treatment groups was performed by histopathological method. A mouse hepatic stellate cell (HSC) model and a mouse liver fibrosis model were constructed to verify the expression of circ_0000623, miR‐351‐5p, and TFEB. Transfected with mRFP‐GFP‐LC3 to detect the autophagic flux of HSCs. We found that 100 mg/kg or 200 mg/kg of anthocyanins could significantly reduce the degree of liver fibrosis in mice. In addition, anthocyanins can inhibit the proliferation, activation, and migration ability of HSCs. circ_0000623 was lowly expressed in mice with liver fibrosis, and anthocyanin treatment could promote its increased expression. Further testing found that anthocyanins could reverse the blocked autophagic flux induced by PDGF or CCL_4_. This effect is achieved by regulating the expression of TFEB by competitive adsorption of miR‐351‐5p. Anthocyanins could treat liver fibrosis by modulating circ_0000623/miR‐351‐5p/TFEB‐mediated changes in HSC autophagic flux.

## Graphical abstract

This study demonstrated for the first time that anthocyanins can participate in the process of liver fibrosis by regulating the autophagy level of hepatic stellate cells. For the first time, we confirmed the significance of mmu_circ_0000623 in the treatment of liver fibrosis with anthocyanins. Anthocyanins could treat liver fibrosis by modulating circ_0000623/miR‐351‐5p/TFEB‐mediated changes in HSC autophagic flux.

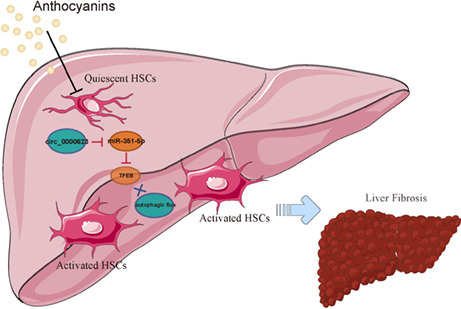

## 1 INTRODUCTION

Liver fibrosis is the abnormal proliferation of fibrous connective tissue in the liver that occurs under the continuous stimulation of liver damage or various chronic inflammations (Kisseleva & Brenner, 2021). It is a pathological change common to all chronic liver diseases. The accumulation of extracellular matrix (ECM) in the liver is mainly increased and the degradation is relatively insufficient, which leads to the occurrence of liver fibrosis after excessive ECM deposition in the liver (Cai et al., 2021; Kisseleva & Brenner, 2021). Liver fibrosis is an intermediate link in the development stage of chronic hepatitis to cirrhosis. Recent studies have shown that liver fibrosis can be reversed, and that liver fibrosis can be reversed at the tissue level if the cause is removed and appropriate drug treatment is given (Dhar et al., 2020; Kumar et al., 2021). Activation of Hepatic stellate cells (HSC) is now recognized as a key event in the progression of hepatic fibrosis (Higashi et al., 2017). Activated HSCs are capable of synthesizing various ECM components, such as type I and collagen III and other proteins that constitute pathological fibrous tissue, leading to the occurrence of liver fibrosis (Higashi et al., 2017). HSCs, also known as lipid storage cells, are mainly found in the hepatic sinuses. Sedentary HSCs are rich in vitamin A, accounting for 15% of the total amount of liver cells. HSC activation can be induced by epithelial cell damage, ECM deposition, immune regulation, membrane receptor, and nuclear receptor‐mediated molecular imbalances, epigenetic‐mediated transcription disorders, cell homeostasis, and stress disorders (Khomich et al., 2019). HSC expression of matrix metalloproteinase and tissue inhibitors of metalloproteinase are responsible for ECM degradation and accumulation, respectively. The imbalance of expression of matrix metalloproteinase and tissue inhibitors of metalloproteinase after HSC activation may be an important factor in the progression of liver fibrosis (Parola & Pinzani, 2019). Studies have shown that the use of safe and effective therapies can delay the progression of cirrhosis and even reverse the stage of liver fibrosis (Zhang et al., 2016). It is worth noting that the regression of hepatic fibrosis is accompanied by a decrease in the number of active HSCs, suggesting that inhibition of HSC activation or elimination of active HSC may be the most effective treatment for hepatic fibrosis.

Blueberry is the common name for the blueberry‐type plant of the Rhododendron family and the bilberry genus (Bingül et al., 2016). According to the USDA research on blueberries, blueberries are rich in protein, fat, carbohydrates, dietary fiber, anthocyanins, and other main components, and have the strong antioxidant capacity (Yan et al., 2019). Therefore, it is listed by the International Food and Agriculture Organization as one of the top five healthy foods for human beings (Yan et al., 2019). Anthocyanins are flavonoid compounds, and current studies have found that they have various pharmacological activities such as antioxidant activity, anticancer, and vision protection (Romualdo et al., 2021; Sun et al., 2018; Wang et al., 2013). The research group previously found that blueberry anthocyanins can regulate the epigenetic changes of hepatic stellate cells, so it may have the effect of treating liver fibrosis (Zhan et al., 2016; Zhan, Liao, Tian, et al., 2017; Zhan, Liao, Xie, et al., 2017).

Circular RNA (circRNA), as one of the members of the human noncoding RNA family, has gradually become the focus of researchers (Jin et al., 2020). CircRNAs are a class of RNA molecules with closed loops, no 5′ cap structure and 3′ end structure, and there are many sources and types (Jin et al., 2020). CircRNAs have a precise location in the cell, mainly concentrated in the cytoplasm and a few in the nucleus. Most circRNAs have highly conserved sequences. Under normal or pathological conditions, they are widely expressed in cells, and their expression abundance is usually linear. The expression of circRNAs in diseases is disease specific and tissue specific; due to the relatively stable structure of a closed loop, circRNAs are not easily affected by nuclease degradation and have a long half‐life. In tumor research, circRNAs had been widely studied; however, there have been few studies in the field of liver fibrosis (Chen & Shan, 2021; Kristensen et al., 2019). Circ_0000623, as a circular RNA, was recently reported to have a low expression in liver fibrosis tissues and was involved in regulating the process of liver fibrosis (Zhu et al., 2020). However, its in‐depth mechanism and whether it plays the same role in the intervention of anthocyanins still need to be determined.

There is currently no research on the mechanism related to anthocyanins and circRNAs. This study is the first to explore the role and mechanisms of circRNAs in treating liver fibrosis with anthocyanins, which can provide a basis for early marker screening and targeted therapy of liver injury.

## 2 METHODS

### 2.1 Experimental animals

Eighty male 8‐week‐old C57BL/6J mice were purchased from Beijing Charles River Laboratory Animal Co., Ltd. All mice were housed with a 12‐h dark–light cycle. They were provided free access to standard diet and water. During the preparation and injection of toxic reagents including CCL_4_, operators should wear personal protective equipment such as glasses, masks, and gloves. All operations were performed in the fume hood and the entire study protocol was reviewed and supervised by the Ethics Committee. The Affiliated Hospital of Guizhou Medical University Animal Ethics Committee approved the experimental protocol, and all experiments were carried out per the Ethical Guidelines for Experimental Animals.

### 2.2 Experimental design

Mice were randomly divided into the following eight treatment groups (10 mice per group): (1) Normal control group (Control): mice were treated with vehicle (10% olive oil, 2 mL/kg, i.p.). The injection dose was 2 mL/kg three times (Monday, Wednesday, and Friday) a week for 12 weeks. (2) CCl_4_ model group: 10% CCl_4_ (2 mL/kg) intraperitoneal injection administered every Monday, Wednesday, and Friday until the end of the 12th week. (3) CCl_4_ + anthocyanins model group (CCl_4_ + Antho 100): During the i.p. injection of CCl_4_, 100 mg/kg body weight of anthocyanins given by gavage every day (Popović et al., 2019; Sun et al., 2018). (4) CCl_4_ + anthocyanins model group (CCl_4_ + Antho 200): During the i.p. injection of CCl_4_, 200 mg/kg body weight of anthocyanins given by gavage every day (Popović et al., 2019; Sun et al., 2018). (5) Control group (Control): mice were treated with vehicle (10% olive oil, 2 mL/kg, i.p.). The injection dose was 2 mL/kg three times (Monday, Wednesday, and Friday) a week for 12 weeks. (6) Normal Control group (NC): 100 μL/mic of AAV8‐NC (1.5 × 10^12^ TU/mL) was injected via the tail vein at the same time as control treatment. (7) CCl_4_ model group: 10% CCl_4_ (2 mL/kg) intraperitoneal injection administered every Monday, Wednesday, and Friday until the end of the 12th week. (8) CCl_4_ model+circ_0000623 group (CCl_4_ + circ_0000623): 100 μL/mic of AAV8‐ circ_0000623 (1.5 × 10^12^ TU/mL) was injected via the tail vein at the same time as CCL_4_ treatment. All animal modeling diagrams are shown in Figure S1. Serum samples were stored at −80°C for further analysis. Liver tissues were excised and stored at −80°C for protein extraction and for histopathology.

### 2.3 Cell culture and treatment

Mice HSC cell line mHSC were purchased from BeNa Culture Collection. It was maintained in RPMI‐1640 medium supplemented with 10% FBS (Clark Bioscience). All cells were placed in a 5% CO_2_ incubator at 37°C after the medium was supplemented with 1% penicillin/streptomycin. They were randomly divided into three groups: control group (medium plus solvent PBS treatment group), PDGF group (20 ng/mL PDGF), and PDGF + anthocyanins group (20 ng/mL PDGF+100 μg/mL anthocyanins), which will be collected mHSC, respectively, for subsequent detection after 48 h of treatment.

### 2.4 Cell proliferation assays

We applied a cell counting kit (CCK‐8; Dojindo) to detect cell proliferation. First, HSCs with different treatments were seeded on 96‐well plates, 10 μL of CCK‐8 solution was added to each cell, and then incubated at 37°C for 1.5 h. Finally, the absorbance was measured at 450 nm using a microplate reader.

### 2.5 Wound‐healing assay

After different treatments, the cells were inoculated into six‐well Petri dishes and cultured for 48 h under appropriate conditions. A sterile pipette tip creates a wound on the cell monolayer. The cells were then washed with PBS and incubated in RPMI‐1640 containing 10% FBS for 48 h. Scratch healing was observed under an inverted microscope at 0 and 48 h, respectively, and was measured by subtracting the posthealing scratch width from the original scratch width.

### 2.6 Autophagy flux detection

mHSCs after different treatments were transfected with mRFP‐GFP‐LC3 (Wanlei bio, China), then the treatment cells were plated on a compartment cover slip, and stained with DAPI for 5 minutes. A confocal laser microscope was used to take pictures of the glass slides of each treatment group.

### 2.7 Western blot analysis (WB)

The mice liver tissue was homogenized with a low‐temperature lysis buffer containing protease inhibitors and phosphatase inhibitors (purchased from ThermoFisher), and cell lysates were prepared in the lysis buffer. After the protein content was determined by the bis‐octanoic acid method (BCA), the same amount of protein was separated from each sample by sodium dodecyl sulfate– polyacrylamide gel electrophoresis (SDS‐PAGE) and transferred to polyvinylidene fluoride PVDF and incubated with the following primary antibodies: Collagen I (1:1000, ab270993), α‐SMA (1:1000, ab7817), TFEB (1:1000, ab264421), LC3 (1:2000, ab192890), P62 (1:1000, ab109012), LAMP1 (1:800, ab208943), LAMP2 (1:500, ab13524), and β‐actin (1:2000, ab8826). After incubating overnight at 4°C with the designated primary antibody, the PVDF membrane was washed with tri‐buffered saline (TBS) (Sigma‐Aldrich) and then incubated with secondary antibody (diluted 1:2000) for 1 hour at room temperature. Finally, the Alphalmager^™^ 2000 imaging system (Alpha Innotech) was used to analyze the gray value of the protein band, and the expression of β‐actin was used as an internal reference.

### 2.8 RNA isolation and real‐time quantitative PCR (qRT‐PCR)

Trizol reagent (ThermoFisher) was used to isolate total RNA from liver tissues or mHSCs and perform reverse transcription. Mir‐X miRNA qRT‐PCR TB Green® Kit (purchased from Clontech) and SYBR Premix Ex Taq II fluorescent quantitative kit (purchased from Takara) were used for a real‐time fluorescent quantitative polymerase chain reaction. The relative level of reverse transcription miRNA and mRNA were normalized based on the relative level of U6 and β‐actin. After PCR amplification, the melting curve of each amplicon was determined to verify its accuracy. All primer sequences are shown in Supplementary Table 1.

### 2.9 Pathological staining

Part of mouse liver tissue was fixed with 10% neutral buffered formalin, embedded in paraffin, and used for histopathological staining. The paraffin‐embedded liver tissue was cut into 4‐μm‐thick sections, and hematoxylin–eosin (H&E) staining (purchased from Beijing Soleibao Technology Co., Ltd.) was then placed under a light microscope to observe the pathological characteristics of liver injury. According to liver fibrosis evaluation criteria, two experienced pathologists assessed the degree of hepatocyte necrosis and inflammatory infiltration. In addition, the sections were stained with Sirius Red (SR) and Masson's trichrome stain to evaluate collagen deposition.

### 2.10 Immunohistochemistry (IHC)

Using methods reported in the literature for reference, immunohistochemistry was used to detect the expression of α‐SMA and col‐I, a classic marker of stellate cell activation and liver fibrosis. Specifically, the embedded liver slices were dehydrated in graded alcohol and then immersed in sodium citrate buffer for 15 min for antigen retrieval. The sections were incubated with α‐SMA and col‐I primary antibody (diluted 1:200) at 4°C overnight. After incubating with the secondary antibody for 30 minutes, sections were placed under a microscope for observation.

### 2.11 Statistical analysis

All data were expressed as mean ± standard error of the mean (mean ± SEM), and GraphPad Prism v9.0 software (GraphPad Software Company) was used for statistical analysis. For the comparison between the two groups that obey the parameter distribution, the unpaired Student's *t*‐test was used. For the nonparametric data that does not obey the parameter distribution, the Mann–Whitney–Wilcoxon nonparametric test was used. *p* < .05 was considered a significant difference.

Enzyme‐linked immunosorbent assay, dual‐luciferase reporter, RNA co‐immunoprecipitation, and other experimental methods are detailed in Appendix S1.

## 3 RESULTS

### 3.1 Anthocyanins attenuate liver fibrosis progression in mice

**FIGURE 1:**
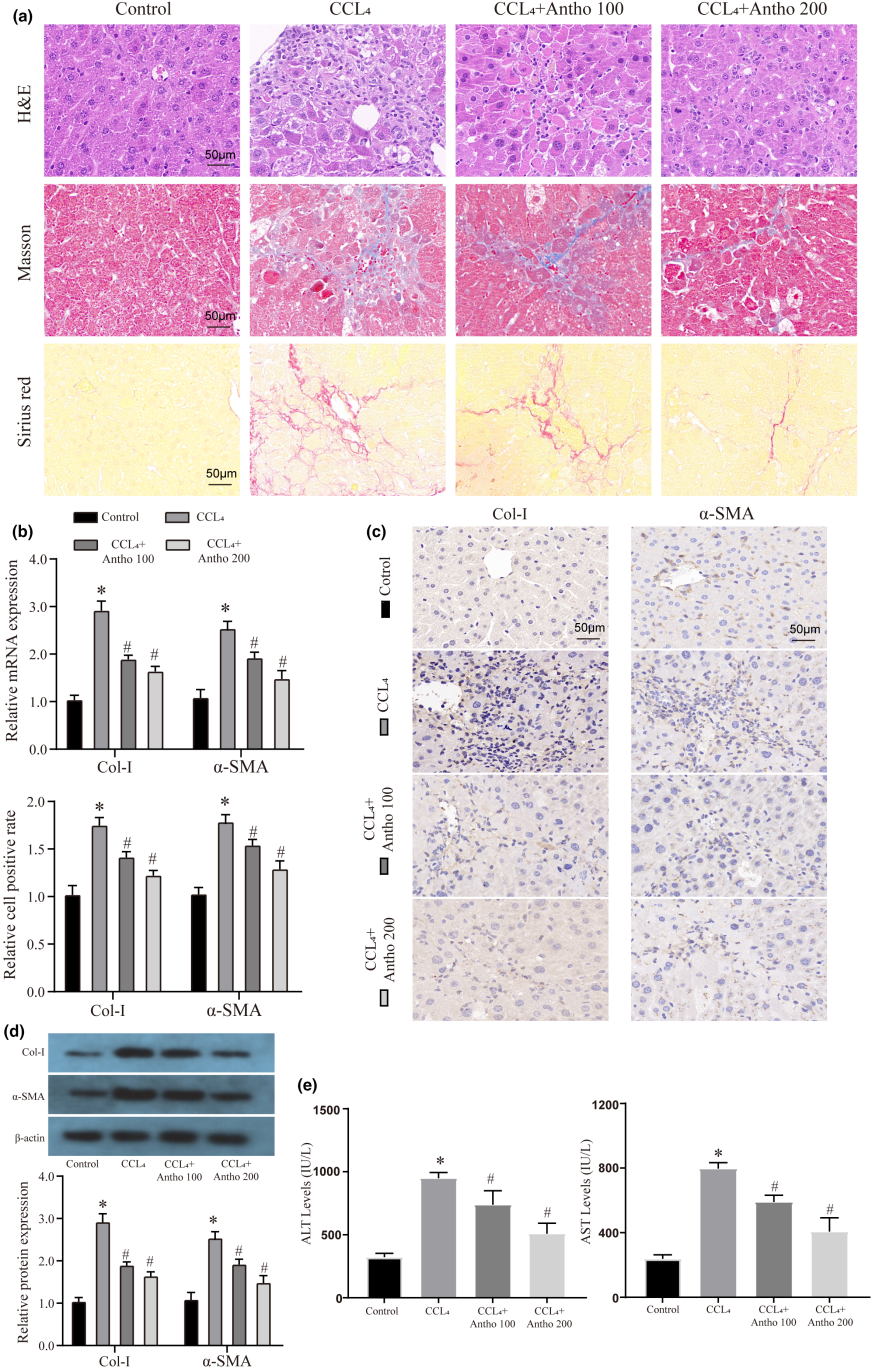
A mouse model of liver fibrosis to verify the therapeutic effect of anthocyanins on the process of fibrosis. (a) HE, Masson, and Sirius red staining verified the effect of different treatment groups on the degree of liver fibrosis in mice. The magnification is 200×. (b) The mRNA expression of liver fibrosis markers (Collagen I and α‐SMA) in different treatment groups was detected by qRT‐PCR. (c) The protein expression of liver fibrosis markers (Collagen I and α‐SMA) in different treatment groups was detected by IHC. (d) The protein expression of liver fibrosis markers (Collagen I and α‐SMA) in different treatment groups was detected by WB. (e) The expression levels of liver injury markers (AST and ALT) in different treatment groups were detected by ELISA. * vs Control group *p* < .05, # vs CCL_4_ group *p* < .05.

First, we used CCL_4_ to construct a mouse liver fibrosis model and administered 100 mg/kg and 200 mg/kg of anthocyanins to the model mice by gavage. We collected the livers of different groups of mice for morphological examination and found that either 100 mg/kg or 200 mg/kg anthocyanins could significantly reduce the degree of liver fibrosis in mice. This phenomenon was evident in Masson staining and Sirius red staining for collagen fibers (Figure 1a). Collagen I and α‐SMA are essential markers of fibrosis, so we detected their mRNA expression and found that anthocyanins could inhibit their expression (Figure 1b). We further verified this result by IHC and WB, respectively (Figure 1c,d). Finally, we found that two doses of anthocyanins could also reduce the expression of ALT and AST in the indicators of liver injury, which indicated that they alleviated the process of liver injury caused by CCL4 (Figure 1e). In addition, to verify the effects of anthocyanins on other organs, we also examined the heart and kidney tissues. Our data show that anthocyanins cannot be used to cause any damage to either organ. This further demonstrates their safety in the treatment process (Figure S2).

### 3.2 Anthocyanins inhibit the hepatic inflammatory response and macrophage polarization

**FIGURE 2:**
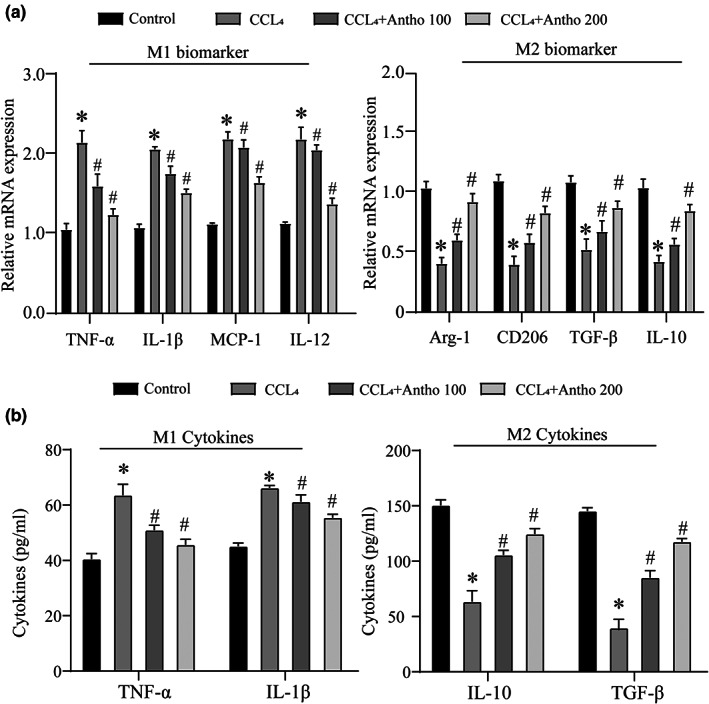
Effects of anthocyanins on inflammatory response. (a) The mRNA expression of macrophage polarization markers in different treatment groups was detected by qRT‐PCR. (b) ELISA detected the protein expression of macrophage polarization markers in different treatment groups. * vs Control group *p* < .05, # vs CCL_4_ group *p* < .05.

As a crucial immune cell in the liver, Kupffer cells are also macrophages in the liver. Studies have confirmed that their polarization degree has a tremendous regulatory effect on liver fibrosis and inflammatory response (Wang et al., 2021). Therefore, we tried to explore the regulatory effect of anthocyanins on its polarization process. First, we verified the expression of M1/2 markers by qRT‐PCR and found that anthocyanins significantly inhibited M1 polarization and promoted M2 polarization (Figure 2a). We further detected the expression of inflammatory factors in the two processes and found that anthocyanins significantly inhibited the proinflammatory process caused by liver fibrosis and promoted the anti‐inflammatory response (Figure 2b). These data suggest that anthocyanins significantly inhibited the inflammatory response and the M1‐type polarization of macrophages during the progression of liver fibrosis.

### 3.3 Anthocyanins inhibit the activation and migration of hepatic stellate cells

**FIGURE 3:**
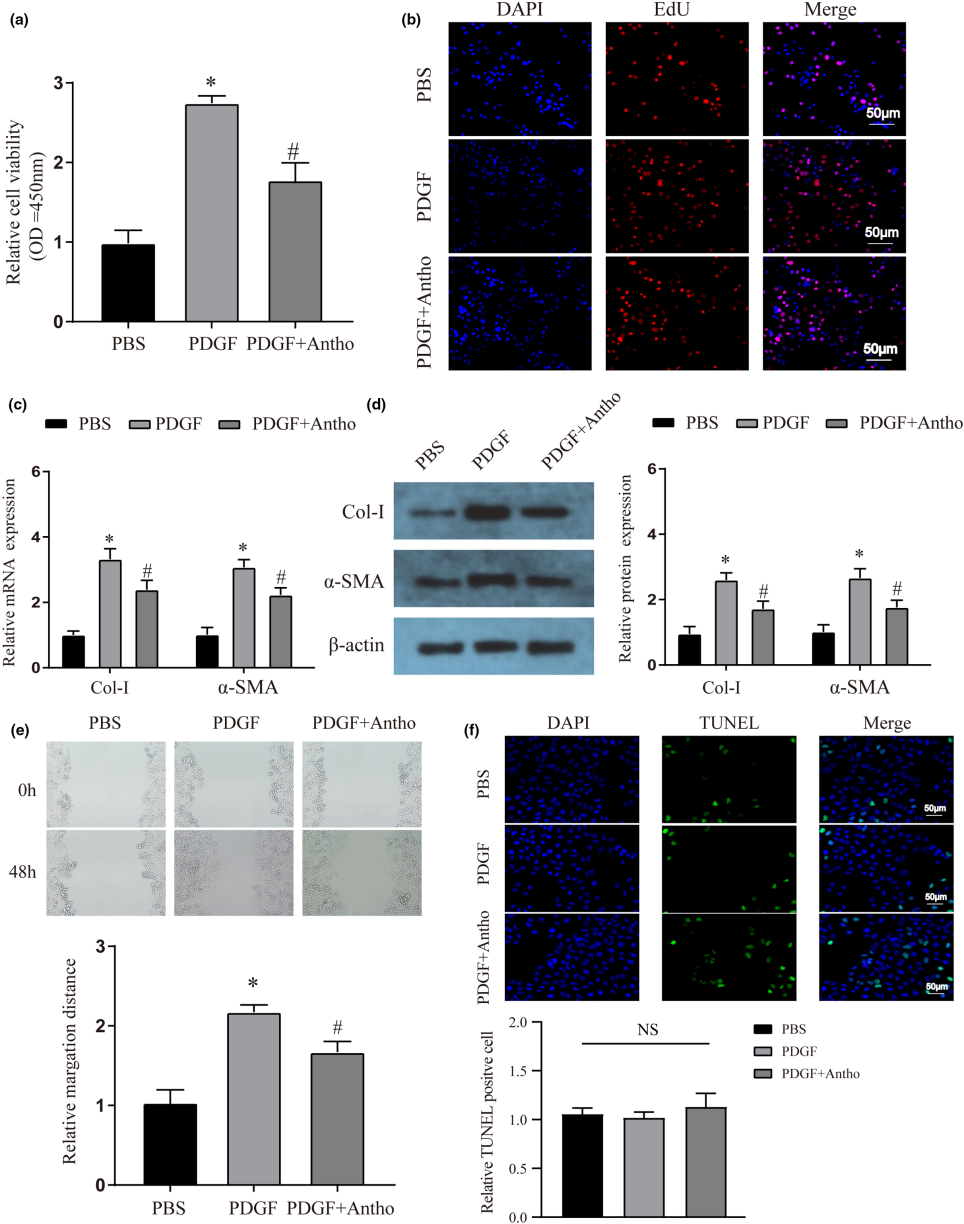
The effects of anthocyanins on HSC proliferation, activation, migration, and apoptosis. (a) CCK‐8 was used to detect the cell proliferation of HSCs after different treatments. (b) EdU staining was used to detect the cell proliferation of HSCs after different treatments. The magnification is 200×. (c) The mRNA expression of liver fibrosis markers (Collagen I and α‐SMA) in different treatment groups was detected by qRT‐PCR. (d) The protein expression of liver fibrosis markers (Collagen I and α‐SMA) in different treatment groups was detected by WB. (e) Wound‐healing assays were used to verify the effect of different treatments on the migration ability of HSCs. The magnification is 100×. (f) TUNEL staining was used to detect the cell apoptosis of HSCs after different treatments. The magnification is 200×. * vs PBS group *p* < .05, # vs PDGF group *p* < .05.

HSCs were the main effector cells in the process of liver fibrosis, and their activation and migration could affect the process of liver fibrosis (Tsuchida & Friedman, 2017; Zhang et al., 2016). Therefore, we examined the effect of anthocyanins on HSC activation, migration, and apoptosis. First, we used 20 ng/mL platelet‐derived growth factor (PDGF) to activate HSCs and detected the proliferation of HSCs by CCK8 and EdU staining. It was found that the treatment group with anthocyanins could inhibit the proliferation of HSCs induced by PDGF (Figure 3a,b). As mentioned above, collagen I and α‐SMA are also markers of HSC activation, and we verified that anthocyanins inhibited their expression by qRT‐PCR and WB, respectively (Figure 3c,d). The migration ability of HSCs is an essential regulatory indicator of the process of liver fibrosis. We used wound‐healing assays to verify that, similar to the proliferation phenomenon, anthocyanins inhibited the migration of HSCs (Figure 3e). Finally, we used Tunel staining to detect the apoptosis of HSCs and found that although the number of positive cells increased in the anthocyanin group, there was no statistical difference in this increase among the groups (Figure 3f). This suggests that anthocyanins may not damage HSC cells to apoptosis.

### 3.4 The role of autophagic flux in the inhibition of HSC activation by anthocyanins

**FIGURE 4:**
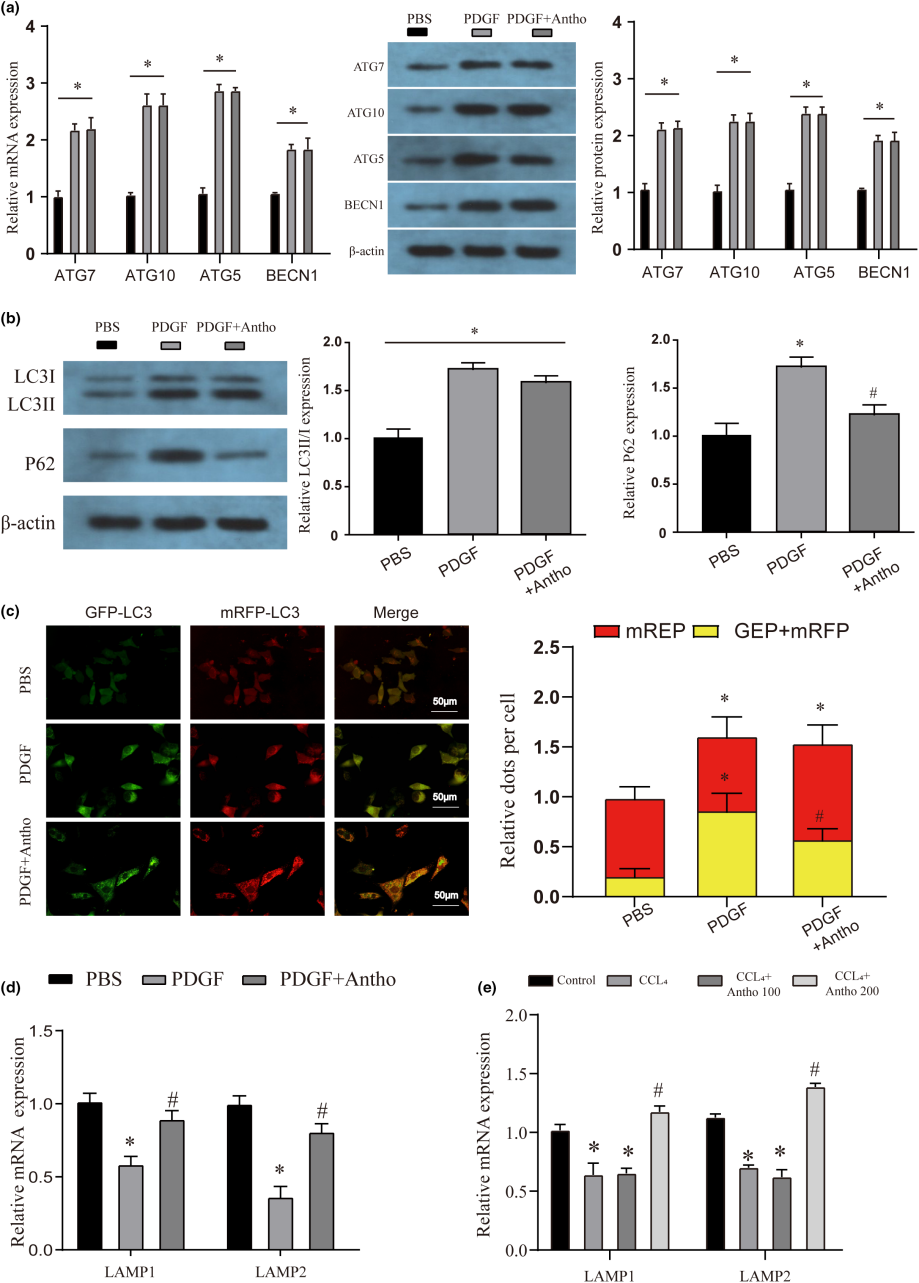
Regulatory effects of anthocyanins on HSC autophagic flux. (a) The mRNA and protein expression of autophagy markers (PINK1, PRKN, ATG5, and BECN1) in different treatment groups was detected by qRT‐PCR and WB. (b) The protein expression of autophagy markers (LC3 and P62) in different treatment groups was detected by WB. (c) The mRFP‐GFP‐LC3 assay detection of autophagy flux in each treatment group. The magnification is 200×. (d) The mRNA expression of lysosome markers (LAMP1 and LAMP2) in HSC of different treatments was detected by qRT‐PCR. (e) The mRNA expression of lysosome markers (LAMP1 and LAMP2) in liver tissues of different treatments was detected by qRT‐PCR. * vs Control or PBS group *p* < .05, # vs CCL_4_ or PDGF group *p* < .05.

Previous studies have shown that changes in autophagy levels can regulate the activation of HSCs and thereby regulate liver fibrosis (Gao et al., 2020). Therefore, firstly, we detected several common autophagy markers and found that autophagy levels were significantly increased in both PDGF‐treated and anthocyanin‐treated groups compared with the PBS group (Figure 4a). We further detected the expression of LC3 protein and P62 protein. We found that the P62 protein in the anthocyanin‐treated group was significantly lower than that in the PDGF group, which may indicate that the early autophagosome formation of HSCs was not affected by PDGF treatment or combined treatment (Figure 4b). However, autophagosomes in the PDGF group were not effectively degraded. To test our hypothesis, we transfected mRFP‐GFP‐LC3 to observe the process of autophagic flux and found that, as expected, autophagy was blocked in the PDGF group, which was manifested by mRFP not being influential in an acidic environment. Degradation increases the number of yellow spots (Figure 4c). Therefore, this study also detected lysosomal markers and found that PDGF significantly disrupted the expression of LAMP1 and LAMP2, inhibited the formation of autophagic lysosomes, and blocked autophagic flux (Figure 4d). This phenomenon was equally demonstrated in an in vivo model (Figure 4e).

### 3.5 The role of TFEB in the regulation of lysosomal function by anthocyanins

**FIGURE 5:**
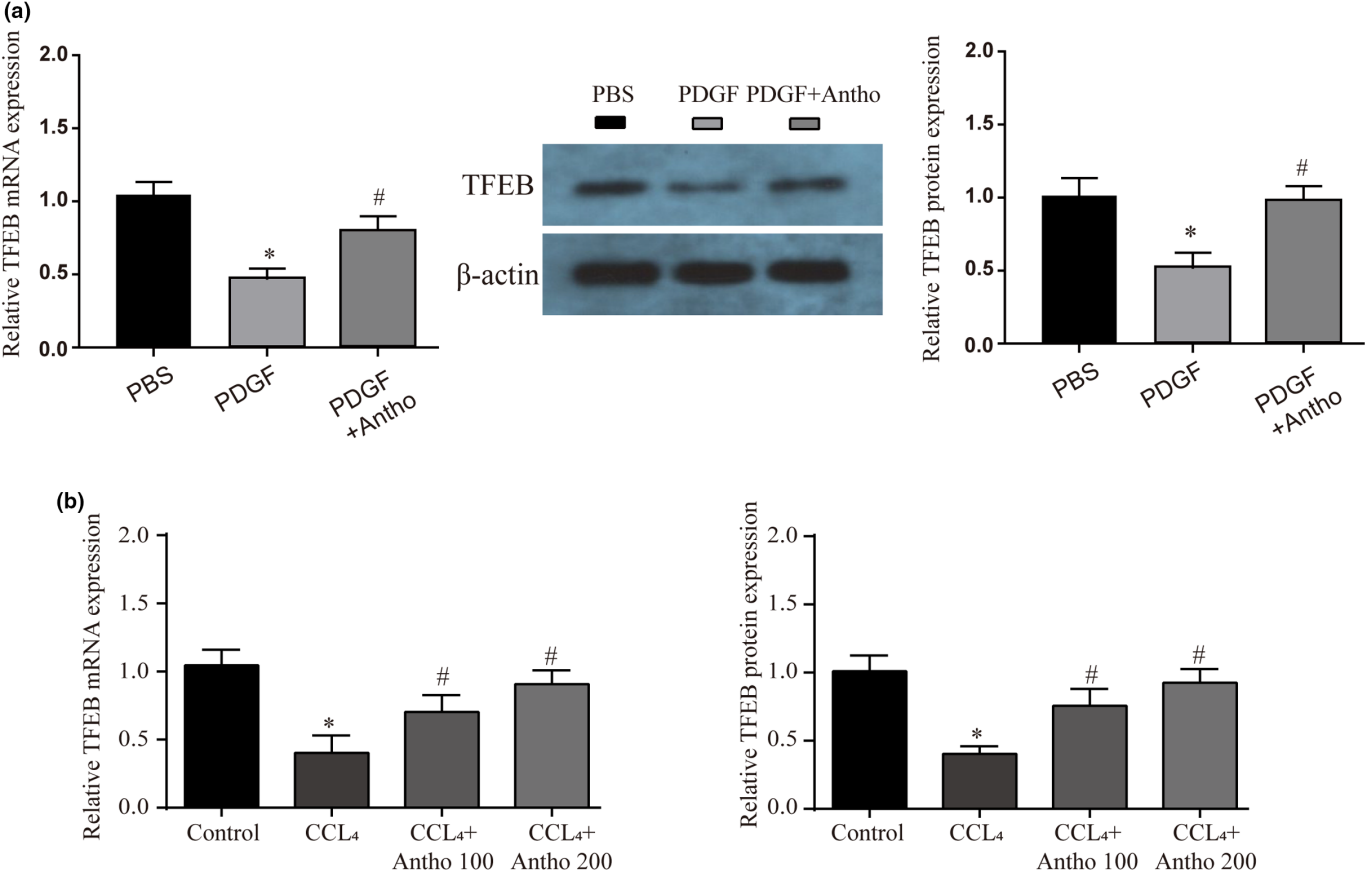
Effects of anthocyanins on TFEB mRNA and protein expression levels. (a) The mRNA and protein expression of TFEB in HSC of different treatments was detected by qRT‐PCR or WB. (b) The mRNA and protein expression of TFEB in liver tissues of different treatments was detected by qRT‐PCR or WB. * vs Control group *p* < .05, # vs CCL_4_ group *p* < .05.

Existing studies have confirmed that TFEB (transcription factor EB) is a significant regulator of lysosomal biosynthesis and autophagy. This role has been demonstrated in alcoholic liver injury and cardiovascular diseases (Alesi et al., 2021; Chao et al., 2018; Wang et al., 2020). In addition, studies have confirmed that its high expression can treat liver damage (Jia et al., 2019a). Therefore, TFEB may play an essential role in the regulation of autophagic flux by anthocyanins. First, we found the same phenomenon as expected in the HSC model, and anthocyanins did increase the expression of TFEB (Figure 5a). This result was similar in our mouse liver (Figure 5b). This suggests that TFEB also plays a vital role in our study.

### 3.6 The role of Circ_0000623/miR‐351‐5p in the regulation of HSC activation and autophagic flux by anthocyanins

**FIGURE 6:**
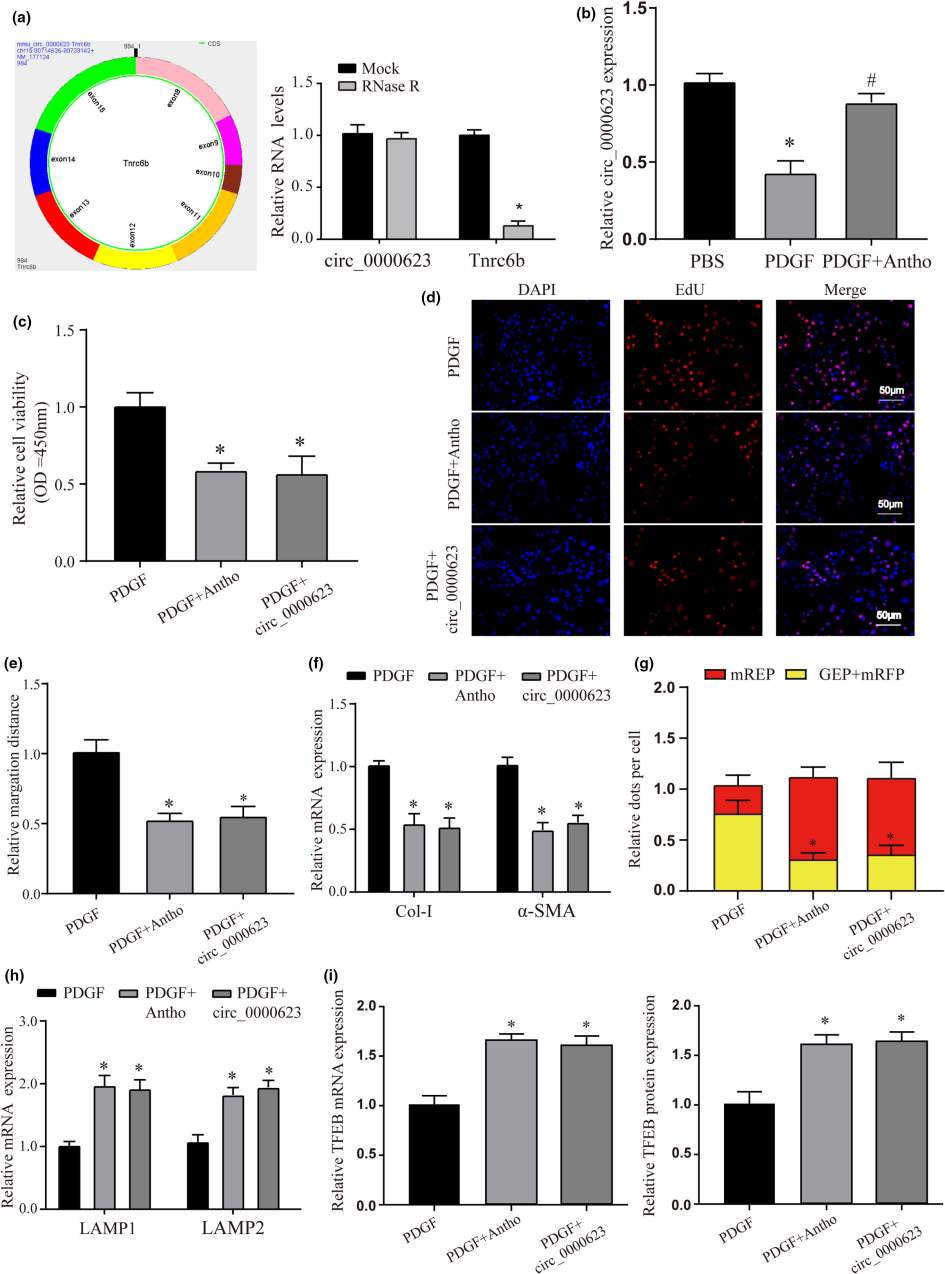
Expression and effect of circ_0000623 after anthocyanin treatment. (a) The loop structure of Circ_0000623 and the expression levels of circ_0000623 and Tnrc6b after RNase R treatment. (b) The circ_0000623 expression in different treatment groups was detected by qRT‐PCR. (c) CCK‐8 was used to detect the cell proliferation of HSCs after different treatments. (d) EdU staining was used to detect the cell proliferation of HSCs after different treatments. The magnification is 200x. (e) Wound‐healing assays were used to verify the effect of different treatments on the migration ability of HSCs. The magnification is 100×. (f) The mRNA expression of liver fibrosis markers (Collagen I and α‐SMA) in different treatment groups was detected by qRT‐PCR. (g) The mRFP‐GFP‐LC3 assay detection of autophagy flux in each treatment group. The magnification is 200x. (H) The mRNA expression of lysosome markers (LAMP1 and LAMP2) in HSC of different treatments was detected by qRT‐PCR. (i) The mRNA and protein expression of TFEB in HSC of different treatments was detected by qRT‐PCR or WB. * vs PBS or PDGF group *p* < .05, # vs PDGF group *p* < .05.

As a highly conserved noncoding RNA, circRNA is a novel biomarker with great research value due to its characteristics. However, there are very few reports of circRNAs in the study of liver fibrosis, and there is no study on anthocyanins. Therefore, we selected circ_0000623 as the research object of this study. First, we verified its circular properties, our data show that RNase R does not degrade it compared to its parent gene (Figure 6a). This proves its loop‐forming ability.

At the same time, we detected the expression of circ_0000623 in HSCs after different treatments and found that the expression of circ_0000623 was significantly reduced after PDGF treatment. Anthocyanins reversed this phenomenon (Figure 6b). To verify the role of circ_0000623 in HSC regulation, we constructed a circ_0000623 overexpression model and used the same method as above to detect the proliferation ability of HSCs. Compared with the PDGF group, both anthocyanins and overexpression of circ_0000623 could inhibit the proliferation of HSCs (Figure 6c,d). We further examined their effects on the expression of migration and fibrosis markers and found that the inhibitory effects of overexpression of circ_0000623 and anthocyanin treatment were similar (Figure 6e,f). This phenomenon is also manifested in the formation of autophagolysosomes (Figure 6g,h). Finally, we tried to explore the role of TFEB, and we found that overexpression of circ_0000623 could also promote the expression of TFEB (Figure 6i). These data validate our hypothesis about the role of circ_0000623 in regulating HSC progression by anthocyanins.

**FIGURE 7:**
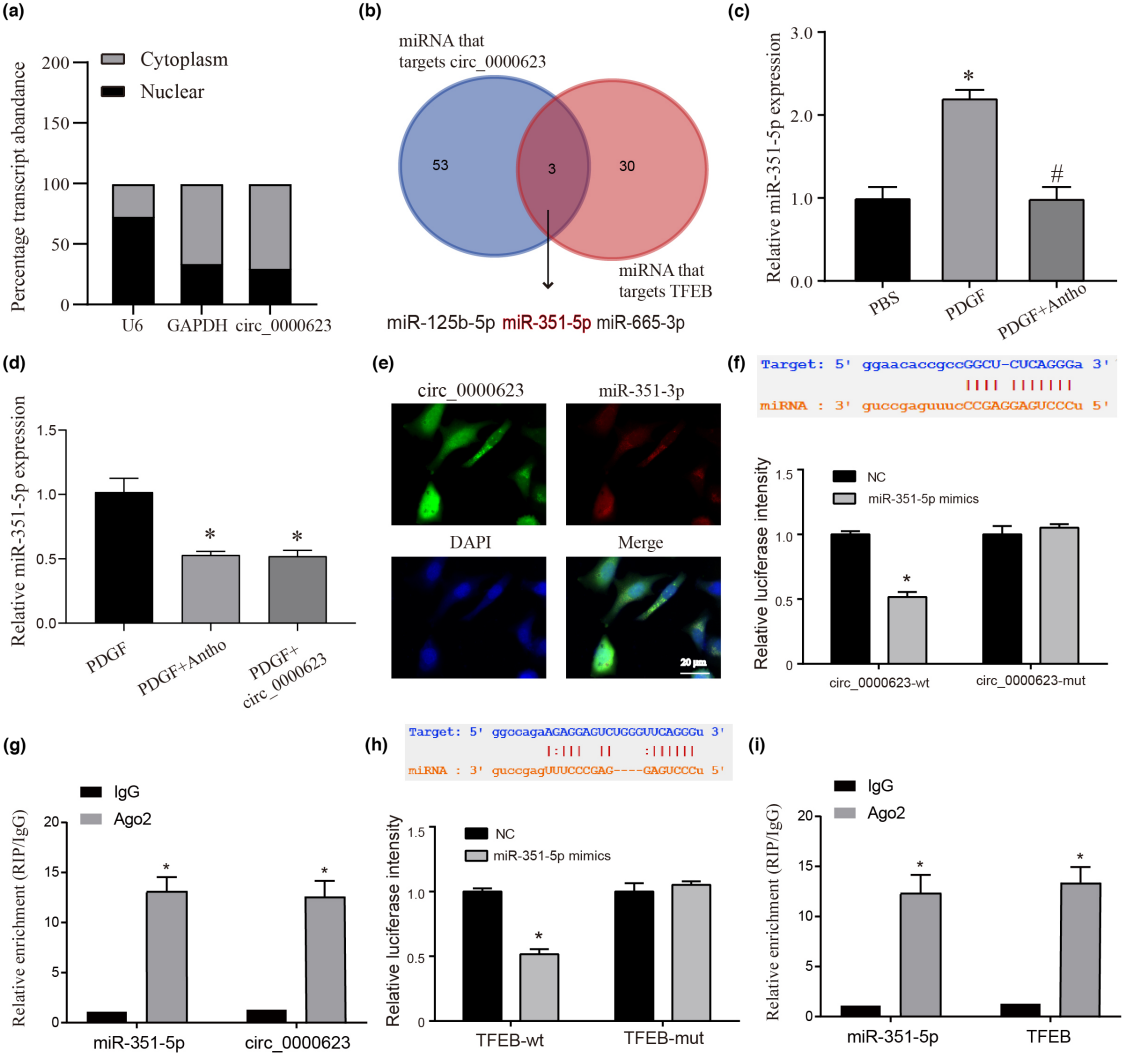
Verification of the targeting relationship among circ_0000623, miR‐351‐5p, and TFEB. (a) QRT‐PCR analysis of subcellular circ_0000623 expression in the nucleus and cytoplasm of mHSC. U6 and GAPDH were used as endogenous controls. (b) Venn diagram screens for cotargeting miRNAs. (c) The miR‐351‐5p expression in different treatment groups was detected by qRT‐PCR. (d) The miR‐351‐5p expression in different treatment groups was detected by qRT‐PCR. (e) The FISH assay was used to verify the sublocalization of circ_0000623 and miR‐351‐5p in mHSC. (f) The dual‐luciferase report verified that circ_0000623 directly binds to the 3'‐UTR region of miR‐351‐5p. (g). RIP assays confirmed the binding status between circ_0000623 and miR‐351‐5p in HEK‐293 T. (h) The dual‐luciferase report verified that miR‐351‐5p directly binds to the 3’‐UTR region of TFEB. (i). RIP assays confirmed the binding status between miR‐351‐5p and TFEB in HEK‐293 T. * vs PBS or PDGF group *p* < .05, # vs PDGF group *p* < .05.

**FIGURE 8:**
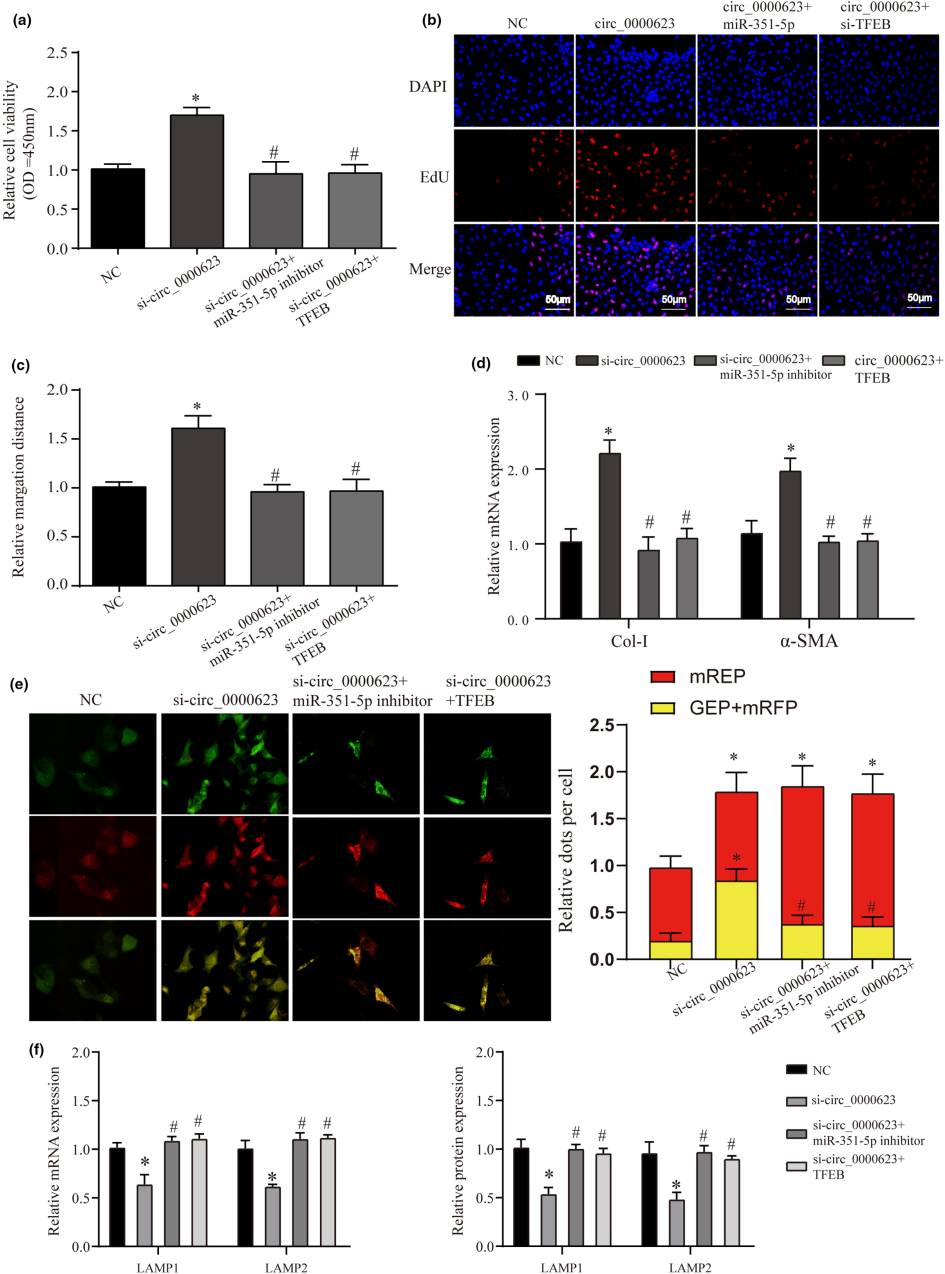
Regulation of circ_0000623, miR‐351‐5p, and TFEB on HSC proliferation, activation, migration, and autophagic flux. (a) CCK‐8 was used to detect the cell proliferation of HSCs after different treatments. (b) EdU staining was used to detect the cell proliferation of HSCs after different treatments. The magnification is 200×. (c) Wound‐healing assays were used to verify the effect of different treatments on the migration ability of HSCs. The magnification is 100×. (d) The mRNA expression of liver fibrosis markers (Collagen I and α‐SMA) in different treatment groups was detected by qRT‐PCR. (e) The mRFP‐GFP‐LC3 assay detection of autophagy flux in each treatment group. The magnification is 200×. (f) The mRNA and protein expression of lysosome markers (LAMP1 and LAMP2) in HSC of different treatments was detected by qRT‐PCR and WB. * vs NC group *p* < .05, # vs si‐circ_0000623 group *p* < .05.

Based on the above data, we found that anthocyanins may play a therapeutic role in liver fibrosis by regulating circ_0000623, but the specific regulatory mechanism of circ_0000623 has yet to be reported. Therefore, we first detected the expression of circ_0000623 in different cellular substructures by nucleocytoplasmic separation and found that it was mainly distributed in the cytoplasm (Figure 7a). We already know that circ_0000623 can play relevant biological roles by competing with resultant miRNAs. Furthermore, our circ_0000623 was able to promote the expression of TFEB. Therefore, we explored the miRNAs of their intermediate roles through the starbase database. A total of three miRNAs were screened by the Venn diagram, namely, miR‐125b‐5p, miR‐351‐5p, and miR‐665‐3p (Figure 7b). We verified the expression of these three miRNAs by overexpressing circ_0000623 and found that the downregulation of miR‐351‐5p was the most significant (Figure S3). Further, we detected the expression of miR‐351‐5p by anthocyanin treatment and found that compared with the PDGF group, anthocyanin could reverse the increased expression of miR‐351‐5p caused by PDGF (Figure 7c). This effect was similar to that of the PDGF+circ_0000623 group (Figure 7d). In addition, we used FISH assays to verify the expression of the two molecules and found that the two molecules colocalized in cells. These data suggest that they have the possibility of targeting results (Figure 7e). Finally, we verified this possibility using dual‐luciferase reporter and RIP, respectively (Figure 7f,g). In addition, we verified the phenomenon of targeted binding between miR‐351‐5p and TFEB in the same way (Figure 7h,i); Taken together from these data, circ_0000623 promotes the expression of TFEB by competitively binding to miR‐351‐5p. In order to further verify the role of this regulatory pathway in regulating HSC activation and autophagy, we first constructed a model of related molecular inhibition or combined processing. First, we verified the expression of model molecules and proved that the expression of related molecules was in line with expectations (Figure S4). Further, we verified the proliferation of HSCs by CCK‐8 and EdU staining. We found that the proliferation of cells in the si‐circ_0000623 group was significantly increased. However, this pro‐proliferation effect was reversed when miR‐351‐5p inhibitor or overexpression of TFEB was added simultaneously (Figure 8a,b).

Furthermore, we found the same phenomenon in migratory ability through wound‐healing assays (Figure 8c). We further examined the expression of fibrosis markers after HSC activation. We found that inhibiting circ_0000623 and simultaneously inhibiting the expression of miR‐351‐5p or inhibiting circ_0000623 and overexpressing TFEB at the same time could reverse the effect of inhibiting circ_0000623 on promoting HSC activation (Figure 8d). They obtained consistent performance in autophagic flux and lysosomal damage (Figure 8e,f). These data confirmed that circ_0000623 could affect HSC activation and migration in vitro through the miR‐351‐5p/TFEB pathway.

### 3.7 In vivo assays to verify the role of circ_0000623 in the treatment of liver fibrosis

**FIGURE 9:**
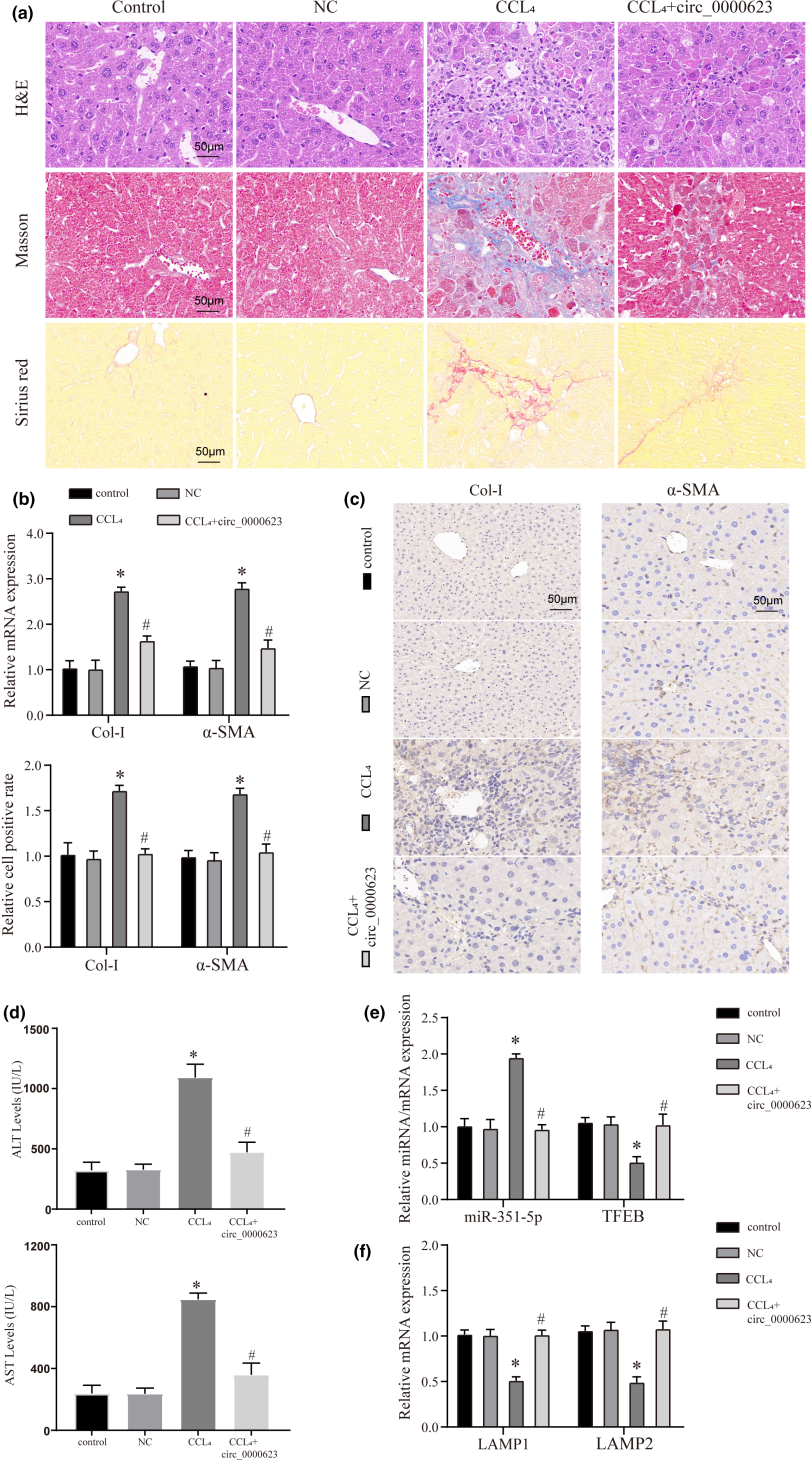
In vivo model to verify the regulatory effect of circ_0000623 on the progression of liver fibrosis in mice. (a) HE, Masson, and Sirius red staining verified the effect of different treatment groups on the degree of liver fibrosis in mice. The magnification is 200×. (b) The mRNA expression of liver fibrosis markers (Collagen I and α‐SMA) in different treatment groups was detected by qRT‐PCR. (c) The protein expression of liver fibrosis markers (Collagen I and α‐SMA) in different treatment groups was detected by IHC. (d) The protein expression of liver fibrosis markers (Collagen I and α‐SMA) in different treatment groups was detected by WB. (e) The expression levels of liver injury markers (AST and ALT) in different treatment groups were detected by ELISA. (f) The mRNA expression of lysosomal damage markers (LAMP1 and LAMP2) in different treatment groups was detected by qRT‐PCR. * vs Control or NC group *p* < .05, # vs CCL_4_ group *p* < .05.

Finally, to verify the role of circ_0000623 in liver fibrosis, we constructed a mouse liver fibrosis model and an in vivo overexpression model. The results of the morphological staining of liver tissue showed that CCL_4_ could promote the process of liver fibrosis compared with the control or NC groups Figure 9a. However, the fibrosis process was significantly improved when circ_0000623 was overexpressed. Further, we detected fibrosis markers by qRT‐PCR and IHC and found that circ_0000623 could inhibit the expression of fibrosis genes and proteins (Figure 9b,c). The associated liver damage was also significantly improved (Figure 9d). Finally, we detected the expression of miR‐351‐5p and TFEB and found that overexpression of circ_0000623 could reverse the promoting effect of CCL_4_ on miR‐351‐5p and the suppressing effect of TFEB (Figure 9e). In addition, we also found that circ_0000623 improves lysosome function in vivo (Figure 9f).

## 4 DISCUSSION

Current studies have found that changes in autophagic flux are an essential factor in HSC activation in the occurrence and development of liver fibrosis (Gao et al., 2020). The abnormal autophagy of HSC often causes HSC cell proliferation, production of a large amount of collagen, imbalance of matrix degradation, enhancement of vasoconstriction, and increase of intrahepatic blood flow pressure, etc., thereby accelerating the process of liver fibrosis (Meng et al., 2018). Previous studies believed that the reversal of liver fibrosis was mainly related to the transition of activated HSCs to a quiescent state; however, other studies have shown that inhibiting HSC activation and proliferation also plays a vital role in treating liver fibrosis (Zhang, Zhou, et al., 2020). Wild blueberries are rich in anthocyanins, which are bioflavonoids with a special molecular structure and are natural antioxidants (Liu et al., 2021; Travica et al., 2020). Our group has previously discovered its role in liver injury (Zhan et al., 2016; Zhan, Liao, Tian, et al., 2017; Zhan, Liao, Xie, et al., 2017). In this study, we further verified that anthocyanins could significantly improve the process of liver fibrosis and liver injury. In addition, we report for the first time that anthocyanins can inhibit liver fibrosis by promoting the expression of circ_0000623 to mediate the miR‐351‐5p/TFEB pathway to regulate changes in HSC autophagic flux.

In this study, we first verified the application of anthocyanins in the field of liver fibrosis in vitro and in vivo. Although previous studies, including our research group, have paid attention to the therapeutic effect of anthocyanins on liver injury, in addition to the mouse liver fibrosis model, this study carried out in vitro investigation of the effect of anthocyanins on HSC proliferation, activation, migration, and apoptosis was further verified. These data further support the prospect of anthocyanins in treating liver fibrosis. However, our results of TUNEL staining showed that anthocyanin treatment did not cause a significant increase in the rate of apoptosis. This suggests that anthocyanin treatment of liver fibrosis is not achieved by promoting HSC apoptosis. Therefore, this also draws our attention to changes in the level of autophagy.

Autophagy can decompose and reuse the organelles, proteins, and macromolecules in cytoplasm to maintain homeostasis, but when autophagy is overactivated, it can lead to suicidal cell death (Gao et al., 2020). Autophagy is tightly regulated by autophagy‐related genes (ATGs, Jia et al., 2019b). When the outside world stimulates cells, autophagic vacuoles are first formed in the cells, and then ATG5 complexes are formed and fused with autophagic vacuoles (Tong et al., 2021; Zhang, Guo, et al., 2020; Zhou et al., 2021). The soluble form (LC3‐I) is converted into the lipid‐soluble form (LC3‐II), which combines with autophagosomes to form autophagosomes (Mohamed et al., 2019). Our data suggest that neither anthocyanin nor PDGF‐treated HSCs had an effect on the formation of early autophagic vacuoles, and both promoted their formation. However, we found that the expression of P62 protein in the PDGF group was higher than that in the group added with anthocyanin, indicating that the autophagic degradation might be inhibited. This was also confirmed in our later fluorescent assay of autophagy flux. Therefore, we verified the function of lysosomes and found the reason for the blockage of autophagy. In addition, many studies have confirmed that TFEB regulates lysosome generation, so we detected its expression and found abnormalities. Therefore, our data can find that anthocyanins can improve the blocked autophagic flux caused by liver fibrosis by regulating the expression of TFEB.

With the continuous development of biomedical research methods in recent years, the function of competing for endogenous RNA (ceRNA) has received more and more attention. In the study of ceRNA, the function and mechanism of circRNA have attracted the attention of researchers. CircRNAs play an essential regulatory role in the occurrence and development of liver development, liver fibrosis lesions, and liver fibrosis‐related liver diseases but also are expected to become biological markers and therapeutic targets for related liver diseases. Related research on circRNA provides a vital research basis for the development of targeted drugs for circRNA‐related targets in clinical practice and the clinical diagnosis and diagnosis of related diseases (Jin et al., 2020; Xu et al., 2022; Zhu et al., 2020). Although the development of circRNA‐related targeted drugs is not yet mature, many studies have revealed the potential of circRNAs as targeted drugs. For example, circ‐SMARCA5 can target miR‐767‐5p to inhibit the progression of multiple myeloma, and hsa_circ_0002483 can target miR‐182‐5p to inhibit the progression of non‐small‐cell lung cancer (Li et al., 2019; McCaw et al., 2019). Although a large number of circRNAs are involved in the regulation of liver development, liver fibrosis, and liver fibrosis‐related liver diseases, the number of circRNAs that have been explored for the relevant mode of action and mechanism is very limited (Jin et al., 2020; Xu et al., 2022; Zhu et al., 2020). Therefore, the regulatory role of circRNAs in liver development, liver fibrosis, and liver fibrosis‐related liver diseases remains to be further explored. The mechanism of action of circRNAs and their roles in related regulatory networks still have significant research value.

Zhu et al found that mmu_circ_0000623 derived from adipose‐derived mesenchymal stem cell exosomes could prevent liver fibrosis (Zhu et al., 2020). Therefore, we sought to verify whether circ_0000623 played the same role in anthocyanins and anthocyanin‐induced changes in autophagic flux. Our results showed that the expression of circ_0000623 was significantly decreased in both fibrotic mouse livers and PDGF‐treated HSCs, whereas the addition of anthocyanins significantly restored its expression. Further, we found that circ_0000623 could produce similar therapeutic effects to anthocyanins by overexpressing circ_0000623. This suggests that circ_0000623 may be the central effector molecule of anthocyanins in the treatment of liver fibrosis, a finding that was reported for the first time. Therefore, to further verify its in‐depth mechanism of action, we screened the miRNAs between circ_0000623 and TFEB through the database and found three possible molecules (miR‐125b‐5p, miR‐351‐5p, and miR‐665‐3p).

Further, we used the circ_0000623 overexpression model to verify that miR‐351‐5p may be the molecule we need to look for.

Moreover, the findings of He et al. also confirmed that miR‐351‐5p might play an essential role in the process of liver fibrosis, but he did not conduct in‐depth verification of its mechanism (He et al., 2018). This part of the data was confirmed in our in vitro and in vivo studies, and the reasons for the changes in miR‐351‐5p expression were also found. Our extensive in vitro and in vivo data confirmed that anthocyanins inhibit HSC activation and migration by regulating autophagic flux through circ_0000623/miR‐351‐5p/TFEB. However, we still lack enough clinical data to verify the effects of anthocyanins and circ_0000623 molecules. This is also one of the important directions for our research group in future research.

In conclusion, this study found for the first time that anthocyanins could inhibit HSC activation by promoting the expression of circ_0000623, improve lysosomal function, and promote autophagic flux to treat liver fibrosis. This effect may be achieved by circ_0000623 by regulating the expression of the miR‐351‐5p/TFEB pathway.

## AUTHOR CONTRIBUTIONS

**Du jin hui:** Conceptualization (equal); data curation (equal); investigation (equal); methodology (equal); project administration (equal); visualization (equal); writing – original draft (equal); writing – review and editing (equal). **Liu li kun:** Data curation (equal); writing – original draft (equal). **Fan hai qing:** Formal analysis (equal). **Yu yue:** Formal analysis (equal). **Gu fang:** Investigation (equal); software (equal). **Luo yi lin:** Investigation (equal). **yu hui:** Software (equal).

## FUNDING INFORMATION

This study was supported by Science and Technology Project of Guizhou Province (No: [2021] General 038). National Natural Science Foundation of Guizhou Medical University Cultivation Project (No: 19NSP021). Science and Technology Planning Project of GuiZhou Province (No: [2023] Key Project 038).

## CONFLICT OF INTEREST STATEMENT

The authors declare that they have no conflicts of interest.

## ETHICS APPROVAL AND CONSENT TO PARTICIPATE

All animal experiments were approved by the Animal Care and Use Committee of the Ethical Institution of the Affiliated Hospital of Guizhou Medical University. The study followed the Health Guidelines for the Care and Use of Laboratory Animals (National Research Council).

## Supporting information


Appendix S1.

Table S1.

Figure S1.

Figure S2.

Figure S3.

Figure S4.

## Data Availability

The datasets used and/or analyzed during the current study are available from the corresponding author upon reasonable request.
