# Supplementary material for: Anthocyanins improve liver fibrosis in mice by regulating the autophagic flux level of hepatic stellate cells by mmu_circ_0000623
Source: Food Sci Nutr. 2023 May 11;11(6):3002–18. doi: 10.1002/fsn3.3281 (PMC10261807; doi:10.1002/fsn3.3281)
Supplement: Supplementary file 1 — Appendix S1. [file FSN3-11-3002-s006.docx]

**Cell transfection**

TFEB overexpression plasmid (TFEB), miR-351-5p inhibitor, si-circ_0000623 or their negative controls were designed and synthesized by GenePharma (Shanghai, China). mHSCs were transfected with indicated plasmids using Lipofectamine 3000 (Invitrogen). All operations were completed in accordance with the reagent manufacturer's operating procedures.

**Enzyme-Linked ImmunoSorbent Assay (ELISA)**

According to the kit instructions, use the IL-10, TGF-β, TNF-α and IL-1β ELISA kit (Nanjing Jianshe, China) to measure their concentration in the liver tissues by ELISA.

**Dual luciferase reporter assay**

The Starbase database (http://starbase.sysu.edu.cn/) was used to predict the targeted binding site of miR-351-5p on TFEB. The wild-type or mutant TFEB 3'UTR containing the predicted miR-351-5p binding site was PCR amplified and inserted into the pMIR-reporter plasmid. Two luciferase reporter plasmids containing TFEB or circ_0000623 were transfected into cells and miR-351-5p mimics were simultaneously transfected. After 48 hours of incubation, the luminescence was measured using a dual luciferase detection kit (Promega, USA) according to the manufacturer's instructions.

**Ribonucleoprotein immunoprecipitation (RIP) assay**

In short, the cell lysate was blocked with Protein G magnetic beads and incubated with anti-AGO G magnetic beads (Thermo Biotechnology, USA) at 4°C for 1.5 hours. The beads were collected by centrifugation at 700 g for 60S, washed 6 times with RIPA buffer, and resuspended in 50 mmol/L Tris-HCl with a pH of 7.0. Then the magnetic beads were incubated at 70°C for 45 minutes for cross-linking, and then RNA co-IP with anti-AGO antibody was extracted. Finally, the target molecule was quantified by qRT-PCR.

**RNase R treatment**

Total RNA (2 μg) was incubated for 30 min at 37 °C with 3 U/μg of RNase R (Beyotime, China). After treatment with RNase R, the RNA expression level of mmu-circ-0000623 was detected by qRT-PCR.
