# Supplementary material for: Anthocyanins improve liver fibrosis in mice by regulating the autophagic flux level of hepatic stellate cells by mmu_circ_0000623
Source: Food Sci Nutr. 2023 May 11;11(6):3002–18. doi: 10.1002/fsn3.3281 (PMC10261807; doi:10.1002/fsn3.3281)
Supplement: Supplementary file 2 — Table S1. [file FSN3-11-3002-s004.docx]

**Supplementary table 1. Primer sequence**

|  | **Primer Sequence** | |
| --- | --- | --- |
|  | Forward | Reverse |
| mmu_circ_0000623 | 5′- TCCCAGACCTTCCAACCAAA-3′ | 5′- AGTTGTGGGAGGAAGTGCTT-3′ |
| TFEB | 5′- GGTGCAGTCCTACCTGGAGA-3′ | 5′- GTGGGCAGCAAACTTGTTCC-3′ |
| LAMP1 | 5′- GGACAACACGACGGTGACAAG-3′ | 5′- GAACTTGCATTCATCCCGAACTGGA-3′ |
| LAMP2 | 5′- TTCAACACCCACTCCAACTC -3′ | 5′-AGCTGAGCCATTAGCCAAATA-3′ |
| Arg-1 | 5′- CTCCAAGCCAAAGTCCTTAGAG -3′ | 5′-AGCTCTGACCGCAGTGTAAAG-3′ |
| TGF-β | 5′-CTGGATACCAACTACTGCTTCAG-3′ | 5′-TTGGTTGTAGAGGGCAAGGACCT-3′ |
| TNF-α | 5′- GCCTCTTCTCATTCCTGCTTG -3′ | 5′- GGCCATTTGGGAACTTCTCA -3′ |
| IL-1β | 5′-TGCCACCTTTTGACAGTGATG-3’ | 5′-AAGGTCCACGGGAAAGACAC-3′ |
| MCP-1 | 5’- CAGGTCCCTGTCATGCTTCT-3’ | 5’- TCTGGACCCATTCCTTCTTG-3’ |
| IL-12 | 5’- GAGGACTTGAAGATGTACCAG -3’ | 5’- TTCTATCTGTGTGAGGAGGGC -3’ |
| CD206 | 5’- CAAGGAAGGTTGGCATTTGT-3’ | 5’- CCTTTCAGTCCTTTGCAAGC -3’ |
| IL-10 | 5′-GCTCTTACTGACTGGCATGAG-3′ | 5′-CGCAGCTCTAGGAGCATGTG-3′ |
| α-SMA | 5′-GGGAGCAGAACAGAGGAATG-3′ | 5′-CCAAACAAGGAGCAAAGACG-3′ |
| Collagen I | 5′-GCTCCTCTTAGGGGCCACT-3′ | 5′-CCACGTCTCACCATTGGGG-3′ |
| BECN1 | 5′- TTTTCTGGACTGTGTGCAGC-3′ | 5′- GCTTTTGTCCACTGCTCCTC-3′ |
| ATG5 | 5′- TGTGCTTCGAGATGTGTGGTT -3′ | 5′- GTCAAATAGCTGACTCTTGGCAA -3′ |
| ATG7 | 5′- GCTGCTGAGATCTGGGACAT -3′ | 5′- GAGATGTGGAGATCAGGACCAG -3′ |
| ATG10 | 5′- AGGTCAGGGCGAGCGA -3′ | 5′- CCATCGCCTATCTGCTGTGA -3′ |
| MiR-351-5p | 5’- TCCCTGAGGAGCCCTTTGAGCCTG-3’ | 5’- AACGCTTCACGAATTTGCGT -3’ |
| U6 | 5’- CTCGCTTCGGCAGCACA -3’ | 5’- AACGCTTCACGAATTTGCGT -3’ |
| β-actin | 5’- AGTGTGACGTTGACATCCGTA -3’ | 5’- GCCAGAGCAGTAATCTCCTTCT-3’ |
