# Supplementary figures and images for: Anthocyanins improve liver fibrosis in mice by regulating the autophagic flux level of hepatic stellate cells by mmu_circ_0000623
Source: Food Sci Nutr. 2023 May 11;11(6):3002–18. doi: 10.1002/fsn3.3281 (PMC10261807; doi:10.1002/fsn3.3281)

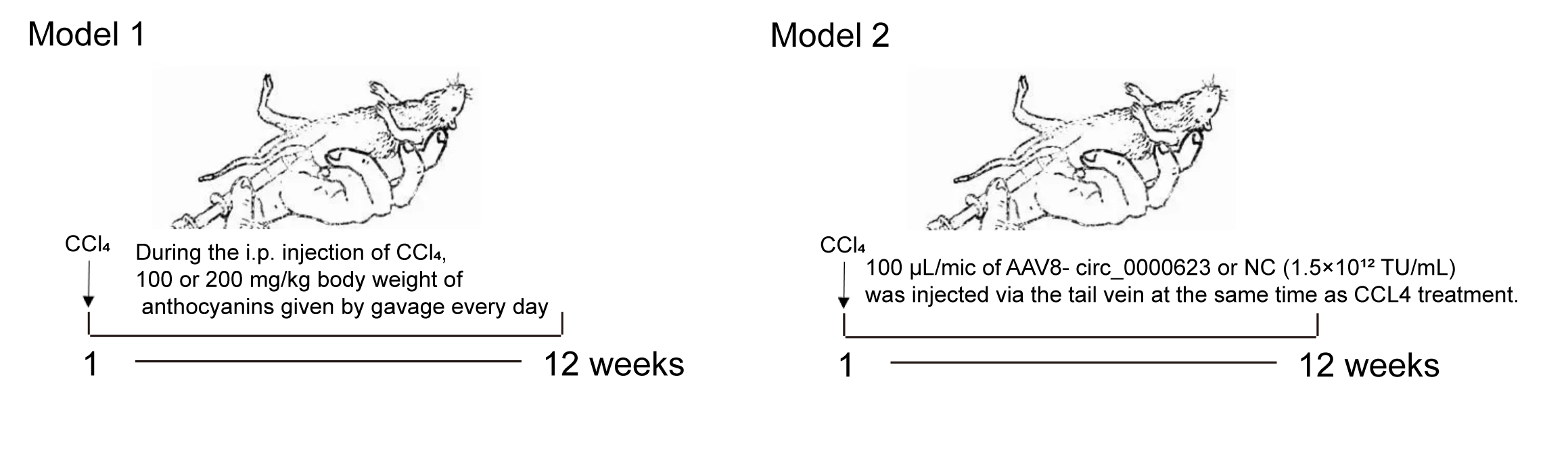

Supplement: Supplementary file 3 — Figure S1. [file FSN3-11-3002-s001.tif]

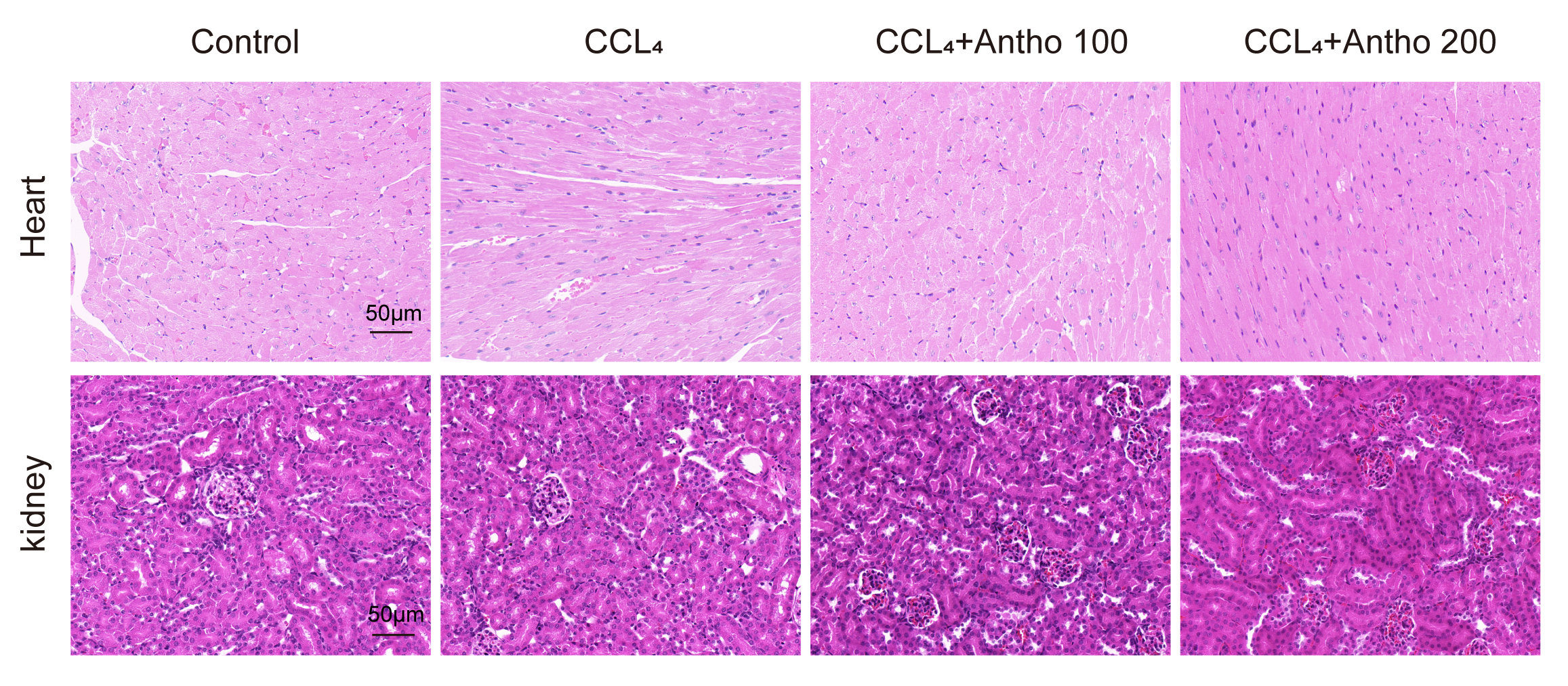

Supplement: Supplementary file 4 — Figure S2. [file FSN3-11-3002-s002.tif]

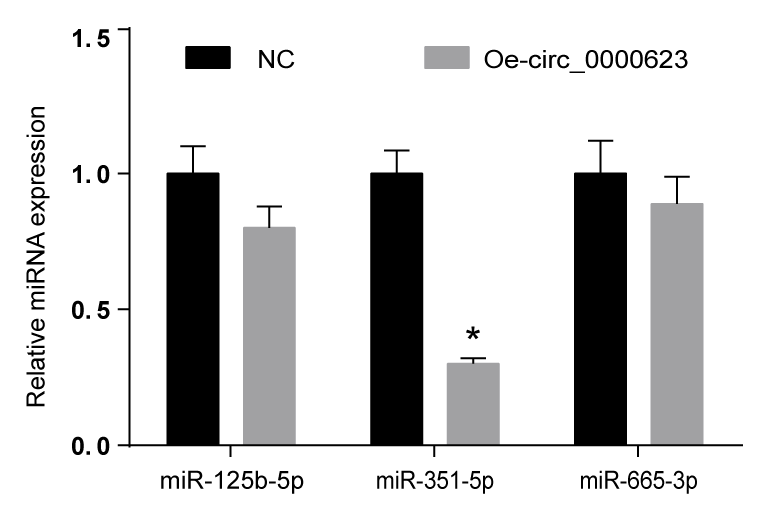

Supplement: Supplementary file 5 — Figure S3. [file FSN3-11-3002-s003.tif]

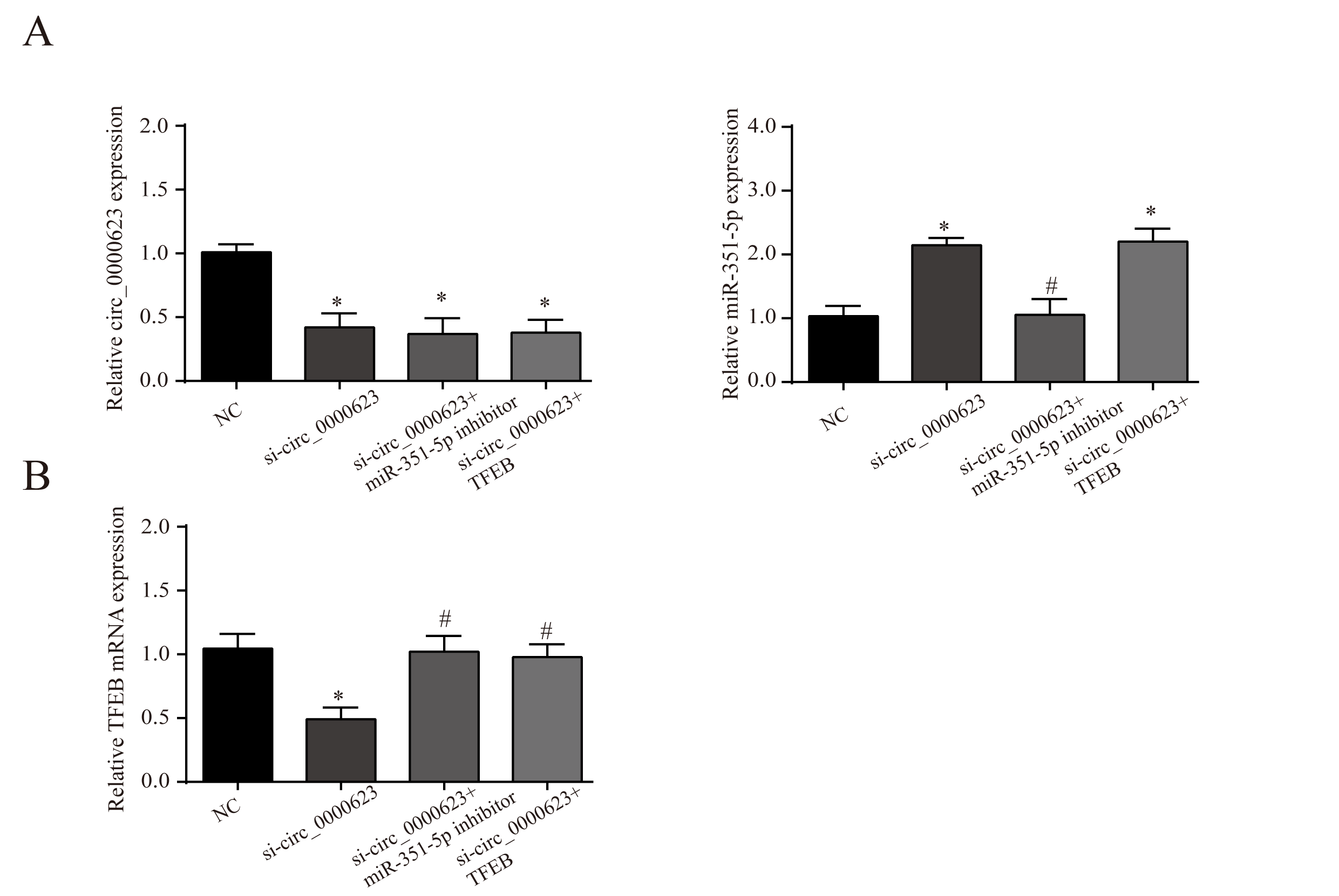

Supplement: Supplementary file 6 — Figure S4. [file FSN3-11-3002-s005.tif]
